# A Highly Sensitive Fluorescent Sensor Based on Carbon Dots and Gold Nanoparticles for Carbaryl Through the Inner Filter Effect

**DOI:** 10.3390/bios15100691

**Published:** 2025-10-13

**Authors:** Yan Lu, Chengqi Bao, Minghui Yang

**Affiliations:** Hunan Provincial Key Laboratory of Micro & Nano Materials Interface Science, College of Chemistry and Chemical Engineering, Central South University, Changsha 410083, China

**Keywords:** fluorescent sensor, carbon dots, gold nanoparticles, inner filter effect, carbaryl

## Abstract

A highly sensitive fluorescent sensing platform was successfully constructed through carbon dots (CDs) and gold nanoparticles (AuNPs) for the specific detection of carbaryl pesticide. Because of the overlap between the fluorescence emission spectrum of CDs and the ultraviolet (UV) absorption spectrum of AuNPs, the fluorescence intensity of CDs exhibited a remarkable decrease in the presence of AuNPs, which was primarily attributed to the inner filter effect (IFE). Acetylcholinesterase (AChE), as a crucial hydrolase in the cholinergic system, can efficiently catalyze the substrate acetylthiocholine iodide (ATChI), leading to the formation of thiocholine. Due to the fact that thiocholine exhibited a positive charge and contained a thiol (-SH), the introduction of thiocholine resulted in the aggregation of AuNPs via gold–thiol bonding and electrostatic interactions. Subsequently, the fluorescence of CDs was restored as the inner filter effect between CDs and AuNPs was alleviated. In addition, carbaryl exerted a significant inhibitory effect on the activity of AChE, impeding the generation of thiocholine and the aggregation of AuNPs, thereby maintaining the fluorescence of CDs quenched. Under the optimal analytical conditions, the detection range of carbaryl is from 0.1 to 200 ng/mL with a detection limit (LOD) of 0.05 ng/mL (S/N = 3). The proposed fluorescent sensor was successfully employed for the detection of carbaryl in strawberry samples with recoveries in the range of 97.5%–101.1%, with the relative standard deviation (RSD) less than 5%.

## 1. Introduction

Carbaryl (1-naphthyl-N-methylcarbamate), as a representative of the carbamate pesticides (CPs), is extensively utilized in agricultural production for the control of insects, mites, and other pests in crops such as fruits, vegetables, and cereals, owing to its high efficiency, low toxicity, and moderate environmental persistence. However, the large-scale and long-term use of carbaryl has led to its widespread residues in agricultural products (e.g., strawberry, cabbage, and wheat), soil, and surface water environments, posing a potential threat to human health and ecological system stability [1,2]. From a toxicological perspective, carbaryl acts as a typical neurotoxin that exerts its toxic effects by targeting acetylcholinesterase (AChE), and the activity of AChE will be inhibited [3,4]. This irreversible inhibition results in the massive accumulation of acetylcholine, leading to neurological dysfunction, respiratory distress, and even direct death of the organism [5]. Hence, it is of great significance in the realm of public health to develop a straightforward, rapid, efficient, and reliable approach for the selective detection of carbaryl residues in various environmental samples.

Different methods have been reported for the detection of pesticides in environmental and food samples [6], such as gas chromatography (GC) [7], high-performance liquid chromatography (HPLC) [8,9], liquid chromatography–mass spectrometry (LC-MS) [10], and surface-enhanced Raman spectroscopy (SERS) [11]. However, these methods suffer from the issues of being time-consuming as well as having a high cost, and they require complex equipment, thus significantly limiting their applications for rapid, on-site detection of CPs [12,13]. In contrast, the fluorescence detection technique has received wide attention due to the advantages of its fast response, high detection sensitivity, and simple operation [14].

With the rapid development of nanotechnology, the application of nanomaterials provides new possibilities for the development of fluorescent sensors with good performances [15]. Different kinds of fluorescent sensors have been designed for the detection of CPs based on organic fluorophores [16], semiconductor quantum dots [17,18], upconversion nanoparticles [19], and carbon nanomaterials [20]. For example, Wu and et al. proposed multifunctional iron-doped polymer dots to detect carbaryl (LOD = 0.8 nM) as the inhibitor of AChE [21]. Mahmoudi and coworkers constructed a proper and reliable fluorometric sensor using carbon quantum dots (CQDs) as the signal reporter for screening AChE and its inhibitors [22]. These fluorescent sensors could be applied to detect pesticide residues indirectly via inhibiting AChE activity. Among these materials, carbon dots (CDs) have garnered increasing attention, considered ideal for the fabrication of fluorescent sensors due to the good optical properties, good biocompatibility, and variable fluorescence properties [23,24,25]. Xu and et al. constructed an effective and simple ratiometric fluorescence sensor by utilizing red-emissive carbon dots (R-CDs) (Em. 677 nm) for carbaryl detection [26].

In the construction of fluorescence sensors, the quenching mechanism highlights several crucial approaches, encompassing photoinduced electron transfer (PET), Förster resonance energy transfer (FRET), intramolecular charge transfer (ICT), and the inner filter effect (IFE) [27]. In addition, gold nanoparticles (AuNPs) play a crucial role in fluorescent sensors [28], and the IFE based on AuNPs and other fluorophores provides an effective strategy in fluorescence sensing [29]. When the emission spectrum of CDs overlaps the absorption spectrum of the quencher, either the IFE or FRET occurs. Compared with FRET, the IFE is unaffected by the distance between the donor and acceptor. In addition, since the IFE is not a static or dynamic quenching process and does not alter the absorption peaks of CDs, it does not affect the fluorescence lifetime of CDs. For instance, Chen and et al. successfully fabricated a fluorescence quenching system based on a silver-modified gold nanorods (Ag^+^ @ AuNCs) for OchratoxinA detection through the IFE [30]. Therefore, the IFE strategy provides a simple alternative method for designing an effective fluorescent sensor [31,32].

**Scheme 1:**
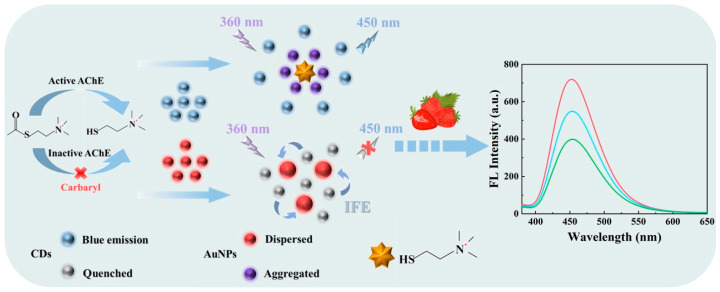
Schematic illustration of the fluorescent sensor based on CDs and AuNPs for carbaryl detection through the IFE.

Herein, a fluorescent sensor for efficient detection of carbaryl was constructed via the IFE between CDs and AuNPs, as shown in Scheme 1. Initially, the fluorescence of CDs was significantly quenched as a result of the IFE in the presence of AuNPs. Thiocholine was obtained through the catalysis of acetylthiocholine iodide (ATChI, an analog of acetylcholine) by AChE. As the surface of thiocholine was positively charged and carries thiol (-SH), in the presence of thiocholine, the uniformly dispersed AuNPs was aggregated through gold–thiol bond (Au-S) interactions and electrostatic interactions. The aggregation disrupted the IFE process, subsequently leading to the restoration of the fluorescence of CDs. However, carbaryl has the potential to inhibit the activity of AChE, thereby preventing the generation of thiocholine and the aggregation of AuNPs. Therefore, in the presence of carbaryl, the fluorescence of CDs was still quenched. Moreover, the sensor has been successfully employed for the detection of carbaryl in real samples.

## 2. Materials and Methods

### 2.1. Reagents and Materials

Carbaryl, gold chloride tetrahydrate (HAuCl_4_·4H_2_O), ethylenediamine (EDA), citric acid (CA), sodium citrate, magnesium chloride (MgCl_2_), sodium sulfate (Na_2_SO_4_), and potassium chloride (KCl) were purchased from Shanghai Aladdin Biochemical Technology Co., Ltd. (Shanghai, China). Acetylcholinesterase (AChE) and acetylthiocholine iodide (ATChI) were obtained from Shanghai Macklin Biochemical Technology Co., Ltd. (Shanghai, China). The phosphate buffer solution (PBS) with varying pH values was prepared through the mixing of 0.01 M potassium dihydrogen phosphate and 0.01 M dipotassium hydrogen phosphate. All chemicals were of analytical grade unless otherwise specified. Ultrapure water with a resistivity of 18 MΩ·cm and a surface tension of 72.6 mN m^−1^ was used throughout the experiments.

### 2.2. Experimental Instruments

Transmission electron microscopy (TEM) images were taken by a JEM-2100 F electron microscope (JEOL, Tokyo, Japan). UV–visible absorption spectra were measured by a UV-2450 spectrophotometer (Shimadzu, Kyoto, Japan). Fluorescence spectra were recorded by an F-7000 spectrofluorometer (Hitachi, Tokyo, Japan) using a xenon lamp. 

### 2.3. Preparation of CDs

The blue-emitting carbon dots were synthesized via a one-step hydrothermal method. Briefly, 10 mmol of CA and 40 mmol of EDA were thoroughly mixed into 50 mL of distilled water and then sonicated for 30 min. After complete dissolution, the mixture was transferred to a 100 mL hydrothermal reactor and heated at 170 °C for 5 h. After the reactor cooled naturally to room temperature, the solution was filtered through a 0.22 μm microporous filter to remove impurities and packed in dialysis bags (MWCO = 1000 Da) for further purification in ultrapure water for 48 h. The final solution was stored at 4 °C in the refrigerator.

### 2.4. Preparation of AuNPs

Prior to the preparation of gold nanoparticles, all the glass instruments were soaked in aqua regia (HNO_3_:HCl = 1:3) and subsequently washed several times with ultrapure water. Subsequently, 100 mL of 0.01% (*w*/*v*) HAuCl_4_·4H_2_O was poured into a three-necked flask, and when heated to boiling, 2 mL of 2% (*w*/*v*) sodium citrate solution was added immediately under vigorous stirring. The reaction was continued for 15 min to obtain a ruby-red AuNP solution. After the reaction was cooled to room temperature, the solution was stored at 4 °C for subsequent experiments.

### 2.5. Fluorometric Detection of Carbaryl

After AChE (10 μL, 1.0 U/mL) and a series of concentrations of carbaryl were first incubated at 37 °C for 15 min, 10 μL of CDs and AuNPs (800 μL, 3.2 nM) and ATChI (10 μL, 1.0 mM) were added to the mixture. The solution was dispensed to 1 mL by using the phosphate buffered solution (pH = 8.0), vibrated and mixed thoroughly, and then incubated for 15 min at 37 °C again. The fluorescence emission spectra were recorded sequentially at 360 nm excitation, and the excitation slit and the emission slit were 5 nm and 5 nm, respectively.

### 2.6. Procedures for the Detection of Carbaryl in Real Samples

To validate the practical utility of the developed fluorescent sensors based on CDs and AuNPs for practical applications, spiking recovery tests were conducted for carbaryl detection in real strawberry samples. Fresh strawberries purchased from the local supermarket were carefully processed by cutting into pieces and grinding to produce a 5 g homogenate, which was then soaked in 25 mL of methanol for 6 h. Subsequently, the mixed solution was centrifuged at 12,000 rpm for 5 min, and the supernatant was collected and filtered through 0.22 μm microporous filter and finally diluted with the phosphate buffer solution (PBS) for the detection of carbaryl in actual samples.

## 3. Results and Discussion

### 3.1. Characteristics of AuNPs and CDs

**Figure 1:**
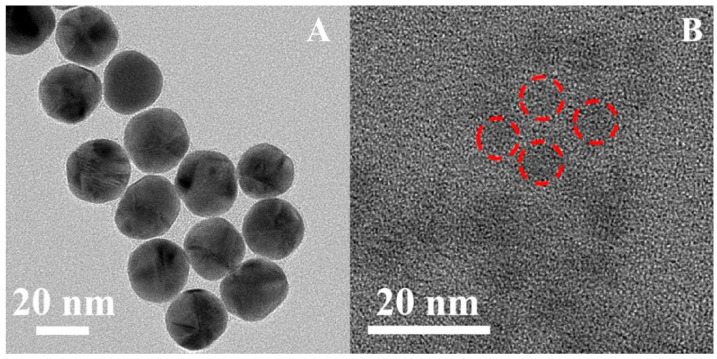
TEM images of (**A**) AuNPs and (**B**) CDs.

Initially, the morphology of CDs and AuNPs was characterized by transmission electron micrographs (TEMs). As shown in Figure 1A, regularly spherical and uniformly dispersed AuNPs with an average particle size of 25 nm were synthesized using sodium citrate as the reducing agent. The CDs also exhibited a spherical shape and uniform dispersion, with an average size of about 5 nm (Figure 1B).

**Figure 2:**
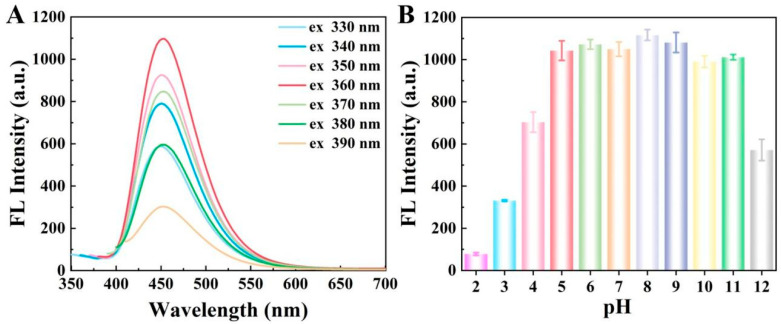
(**A**) Fluorescence emission spectra of CDs at different excitation wavelengths in the range from 330 to 390 nm; (**B**) fluorescence intensity of CDs at different pH values ranging from 2.0 to 12.0 of the phosphate buffer.

After proving the successful synthesis of CDs, the fluorescence properties of CDs were studied. Initially, the fluorescence emission spectra of CDs were recorded at different excitation wavelengths. As illustrated in Figure 2A, the maximum fluorescence intensity was obtained at an emission wavelength of 450 nm when the excitation wavelength was set at 360 nm. These data also demonstrated the successful synthesis of CDs. Then, to study the influence of pH on the fluorescence properties of CDs, the fluorescence intensity of CDs at different pH values was recorded. As depicted in Figure 2B, the fluorescence intensity of CDs remained relatively stable within the pH range of 5.0–11.0, indicting that a pH value in this range has neglectable impact on the fluorescence intensity of CDs, as well as demonstrating good fluorescence stability of CDs.

### 3.2. Fluorescence Quenching Effect Through the IFE Between CDs and AuNPs

**Figure 3:**
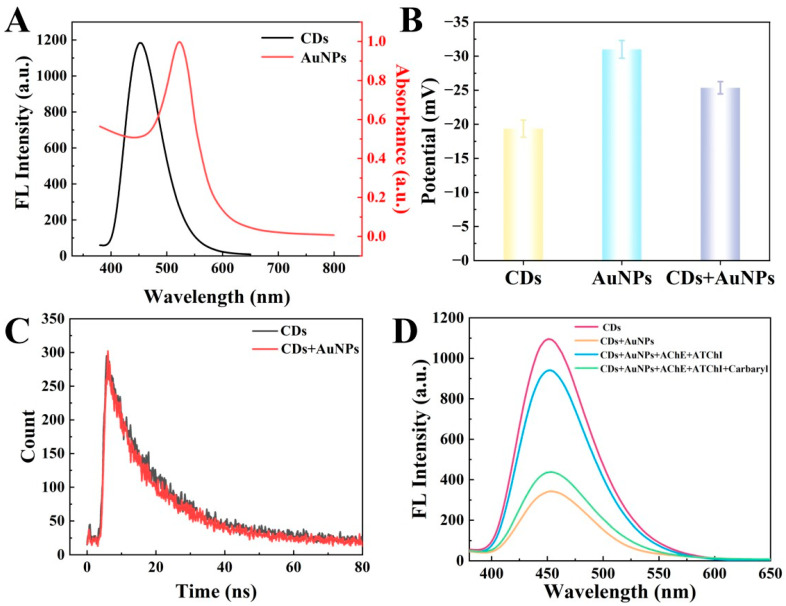
(**A**) UV–vis absorption spectra of AuNPs and fluorescence emission spectra of CDs, respectively; (**B**) the zeta potentials of CDs, AuNPs, and a mixture containing CDs and AuNPs; (**C**) the fluorescence lifetimes decay curves of CDs with AuNPs (red line, τ = 14.6 ns) and without AuNPs (black line, τ = 13.9 ns); (**D**) the fluorescence spectra of CDs, CDs+AuNPs, CDs+AuNPs+AChE+ATChI, and CDs+AuNPs+AChE+ATChI+carbaryl.

To further investigate the fluorescence quenching of CDs by AuNPs, the UV–visible absorption spectra of AuNPs and the fluorescence emission spectra of CDs were measured, respectively. As demonstrated in Figure 3A, the UV–visible absorption spectra of AuNPs exhibited a remarkable overlap with the fluorescence emission spectra of CDs, contributing to the fluorescence quenching effect of AuNPs on CDs and providing an indispensable condition for the inner filter effect (IFE). The fluorescence quenching may be due to the IFE or Förster resonance energy transfer (FRET). However, the photoinduced electron transfer (PET) is an electron transfer process that occurs between CDs and quenchers, during which free radical charge carriers are generated through the excitation of electron receptors or donors. Considering that the FRET process originates from dipole–dipole interactions, the energy transfer between the donor and the acceptor demonstrates a significant reliance on the center-to-center separation distance within 10 nm. Compared to fluorescence methods based on FRET or PET, the IFE requires no electron or energy transfer processes, making it more flexible and straightforward. Fluorescence methods using FRET or PET require a connection between the absorbent and fluorophore, which alters the fluorescence lifetime [31]. Notably, the IFE remains effective even when the distance between the emitter and acceptor exceeds 10 nm. The surface of AuNPs coated with negatively charged citrate ions exhibited a negative zeta potential of −31.0 mV. Meanwhile, as shown in Figure 3B, the zeta potential of CDs also displayed a negative charge at −19.4 mV. Consequently, there existed electrostatic repulsion between CDs and AuNPs, thereby increasing the distance between the two particles. Additionally, both FRET and PET significantly affect the fluorescence lifetime of fluorophores. In contrast, the IFE demonstrates essentially negligible effects on fluorescence lifetime since it does not belong to the static or dynamic quenching process [33]. Consequently, to attain the interaction mechanism between CDs and AuNPs, the fluorescence lifetimes of CDs were determined in the presence or absence of AuNPs. As seen in Figure 3C, in the absence of AuNPs in the system, the fluorescence lifetime of CDs was measured to be 14.6 nanoseconds (ns). However, upon the addition of AuNPs into the system, the fluorescence lifetime of CDs was detected as 13.9 ns. This indicated that the fluorescence lifetime values of CDs remained remarkably constant regardless of whether AuNPs were present or not, demonstrating that the substantial decrease in fluorescence of CDs can be primarily attributed to the IFE rather than FRET or PET.

### 3.3. Feasibility of the Sensor for Detection of Carbaryl

To demonstrate the feasibility of the proposed fluorescent sensor for carbaryl analysis, the performance of the sensor for AChE and the inhibitory effect of carbaryl on AChE were studied. CDs exhibited a strong fluorescence signal at about 450 nm. Once AuNPs were mixed with CDs, the fluorescence intensity of CDs was significantly reduced due to the IFE. Upon the subsequent introduction of AChE and ATChI into the mixed solution containing CDs and AuNPs, the fluorescence intensity was recovered. This phenomenon occurred because the enzymatic reaction between AChE and ATChI generated thiocholine, which effectively interacted with the AuNPs, thereby disrupting the quenching effect on the fluorescence of CDs and leading to the recovery of the fluorescent signal. In addition, upon the addition of carbaryl as an inhibitor of AChE, as expected, the fluorescence recovery of CDs was impeded (Figure 3D). Therefore, the proposed fluorescence sensor was proved feasible for the detection of carbaryl.

### 3.4. Optimization of the Experimental Conditions

**Figure 4:**
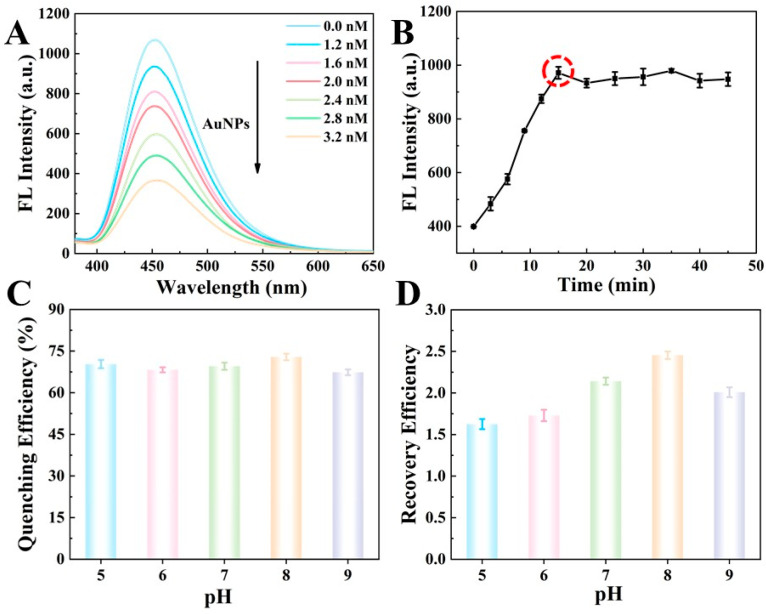
Effects of (**A**) the various concentrations of AuNPs; (**B**) the incubation time between AChE (1.0 U/mL, 10 μL) and ATChI (1 mM, 10 μL) at 37 °C; (**C**) the different pH values of PBS in the range of 5.0–9.0 on the fluorescence quenching efficiency of CDs in the presence of AuNPs; and (**D**) the pH of 0.01 M PBS on the fluorescence recovery efficiency of CDs after AChE/ATChI addition.

To achieve the optimal sensing performance of the fluorescent sensor based on CDs and AuNPs for carbaryl detection, serval crucial parameters were systematically optimized, including the concentration of AuNPs, the incubation time between AChE and ATChI, and the pH of the buffer solution. The effect of the concentration of AuNPs on the fluorescence intensity of CDs was investigated by introducing varying concentrations of AuNPs. As shown in Figure 4A, the fluorescence intensity of CDs decreased with the addition of AuNPs compared with that without AuNPs. Moreover, the fluorescence signal of CDs gradually weakened as the AuNP concentration increased continually. Ultimately, 3.2 nM of AuNPs is selected as the optimal concentration for the assay. When the optimal concentration of AuNPs was 3.2 nM, the quenching efficiency (I_0_ − I_1_)/I_0_ of CDs reached about 73.9%. I_0_ and I_1_ were recorded as the fluorescence intensity of CDs at 450 nm without and with the addition of AuNPs, respectively. For optimal catalytic performance of AChE, the incubation time between AChE and ATChI was studied. AChE (1.0 U/mL, 10 μL) and ATChI (1 mM, 10 μL) were added to a mixture containing CDs and AuNPs. The fluorescence intensity of the mixture was measured every 3 min at 37 °C. As seen in Figure 4B, the fluorescence intensity gradually increased with the incubation time. When the incubation time was 15 min (as shown in the red circle of Figure 4B), the maximum fluorescence signal was obtained and remained stable. Accordingly, 15 min is used as the optimal incubation time for AChE hydrolysis of ATChI in the subsequent experiments. As pH is one of the important factors affecting the catalytic activity of AChE, the effect of the pH of the buffer solution on the fluorescence signal was investigated within the pH range of 5.0–9.0. Initially, in the presence of AuNPs, the quenching efficiency of CDs in 0.01 M PBS at various pH was analyzed. As depicted in Figure 4C, the quenching efficiency remained relatively stable within the pH range of 5.0–9.0, suggesting that fluctuations in pH had a negligible influence on the quenching efficiency. In addition, as shown in Figure 4D, the fluorescence recovery efficiency of the CDs at pH 8.0 achieved the maximum of approximately 2.45, as indicated by the ratio (I_2_ − I_1_)/I_1_. Furthermore, I_2_ was designated to represent the fluorescence signals of CDs after the introduction of AChE and ATChI. As a result, pH = 8.0 is selected as the optimal pH for the buffer solution for the subsequent assay.

### 3.5. Detection of Carbaryl

**Figure 5:**
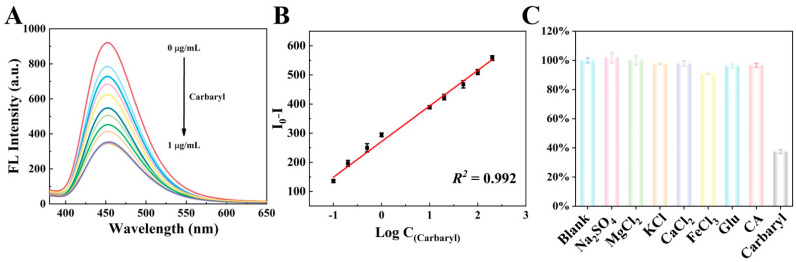
(**A**) Fluorescence emission spectra of the CDs upon the addition of different concentrations of carbaryl in 0.01 M PBS with a pH of 8.0; (**B**) linear relationship between fluorescence quenching of CDs and carbaryl concentration in the range of 0.1–200 ng/mL; (**C**) selectivity analysis of the fluorescent sensor based on CDs and AuNPs for the detection of 200 ng/mL carbaryl.

To comprehensively evaluate the analytical performance of the proposed fluorescent sensor based on CDs and AuNPs for carbaryl detection, the fluorescence signal of CDs was systematically assessed across a range of carbaryl concentrations under optimized experimental conditions. As shown in Figure 5A, the fluorescence emission intensity of CDs at 450 nm gradually decreased with the increase in carbaryl concentration. I_0_ and I were recorded as the fluorescence intensities of CDs at 450 nm without and with the addition of carbaryl to calculate the value of I_0_ − I. Figure 5B indicates that the value of I_0_ − I shows a good linear relationship with the logarithm of the concentration of carbaryl from 0.1 to 200 ng/mL. The linear calibration curve is I_0_ − I = 122.3 log C + 271.2 (*R*^2^ = 0.992). The limit of detection (LOD) of the sensor was calculated to be 0.05 ng/mL based on an S/N value of 3 [26]. The LOD is significantly lower than the maximum residue limits (MRLs) of carbaryl in agricultural products stipulated by regulatory authorities, which indicates the high sensitivity of the sensor for the detection of trace amounts of carbaryl. In addition, the performance of our fluorescent sensor was compared with other reported analytical methods. As shown in Table 1, it can be seen that the developed sensor has a wider linear range and a lower detection limit.

### 3.6. Selectivity Study

The selectivity of the proposed fluorescent sensor was systematically evaluated by measuring the response towards 200 ng/mL of carbaryl in comparison with various potential interfering substrates. Specifically, the responses of the fluorescent sensor toward these interfering substances were investigated by introducing 100-fold concentrations of KCl, Na_2_SO_4_, FeCl_3_, CaCl_2_, MgCl_2_, glucose, and citric acid compared to carbaryl. As depicted in Figure 5C, with the addition of the above substances, only carbaryl resulted in the quenching of fluorescence of CDs, indicating that the activity of AChE was not inhibited by these interfering substances. Therefore, based on the above data, it can be concluded that the fluorescent sensor exhibits high selectivity for carbaryl detection.

### 3.7. Real Sample Analysis

The practical application of the fluorescent sensor was evaluated by a spiking recovery test of different concentrations of carbaryl in strawberry samples. A blank sample without carbaryl was prepared under the same conditions, demonstrating that no detectable traces of carbaryl were present in the system. The different concentrations of carbaryl standard solutions were set at 0.2 ng/mL, 50 ng/mL, and 200 ng/mL. As listed in Table 2, the recoveries ranged from 97.5% to 101.1%, with the relative standard deviation (RSD) less than 5%. The above results indicate that the developed sensor is reliable for the determination of carbaryl in real samples.

## 4. Conclusions

In summary, in this work, a novel fluorescence sensing platform based on carbon dots (CDs) and gold nanoparticles (AuNPs) was successfully established and employed for the selective and sensitive detection of carbaryl residues in the strawberry samples. The fluorescence emission spectra of CDs demonstrated an overlap with the ultraviolet (UV) absorption spectra of AuNPs, consequently triggering a strong IFE that resulted in the efficient quenching of the fluorescence of CDs. Acetylcholinesterase (AChE) efficiently catalyzed the hydrolysis of acetylthiocholine iodide (ATChI), leading to the generation of thiocholine as the primary product. Thiocholine, through specific gold–thiol (Au-S) bonding and electrostatic interactions with gold nanoparticles (AuNPs), triggered the aggregation of AuNPs, which notably attenuated the IFE and consequently promoted the significant recovery of the fluorescence intensity of CDs. In addition, upon the introduction of carbaryl into the system, it exerted a notable inhibitory influence on the enzymatic activity of AChE. As a result, the fluorescence signal of CDs remained quenched. In the carbaryl detection assay, the developed sensor exhibited outstanding linear response characteristics (*R*^2^ = 0.992) within a broad concentration range from 0.1 to 200 ng/mL (LOD = 0.05 ng/mL). It is worth mentioning that the sensor could be successfully applied for the detection of carbaryl in strawberry samples and the recoveries ranged from 97.5% to 101.1%, with the RSD less than 5%, thereby indicating the sensor has promising applications for monitoring carbaryl for safeguarding food safety and the ecological environment.

## Figures and Tables

**Table 1:** Comparison the performance of the proposed sensor with other reported analytical methods for carbaryl pesticide detection.

Material	Method	Linear Range (ng/mL)	LOD (ng/mL)	Ref.
NSAC ^a^	HPLC-UV ^b^	3.5–10,000	0.1	[34]
MWCNTs ^c^-GONRs ^d^	Electrochemical	1–1000	0.34	[35]
e-pGON ^e^	Electrochemical	0.3–6.1	0.15	[36]
CdTe QDs ^f^	Fluorescent	50–14,000	0.12	[37]
MACC ^g^	Fluorescent	4–200	5.81	[38]
NH_2_-MIL-101(Fe) ^h^	Fluorescent	2–100	1.45	[39]
PAT ^i^-CDs@SiO_2_ ^j^	Fluorescent	10–100	3.3	[40]
R-CDs ^k^	Fluorescent	0–20,000	0.52	[26]
CDs+AuNPs	Fluorescent	0.2–200	0.05	This work

^a^ NSAC: ultrasound-assisted dispersive sponge-activated carbon extraction; ^b^ HPLC-UV: high-performance liquid chromatography–ultraviolet; ^c^ MWCNT: multi-walled carbon nanotubes; ^d^ GONRs: graphene oxide nanoribbons; ^e^ e-pGON: the electrochemically inducing porous graphene oxide network; ^f^ QDs: quantum dots; ^g^ MACC: Mn^2+^-activated CRISPR-Cas12a system; ^h^ MIL-101(Fe): Fe-based metal–organic framework; ^i^ PAT: L-aspartic acid and L-tryptophan; ^j^ PAT-CDs@SiO_2_: PAT-CDs embedded in SiO_2_ nanoparticles; ^k^ R-CDs: red-emission CDs synthesized by using taxus, a natural biomass, as a carbon source.

**Table 2:** Determination of carbaryl in strawberry samples (n = 3) using the proposed fluorescent sensor.

Sample	Added (ng/mL)	Found (ng/mL)	Recovery (%)	RSD (%)
1	0	-	-	-
2	0.20	0.195	97.5%	1.09
3	50.0	50.54	101.1%	4.93
4	200	199.3	99.7%	4.76

## Data Availability

The original contributions presented in this study are included in the article. Further inquiries can be directed to the corresponding author.

## References

[1] Adhikari S., Joshi R., Joshi R., Kim M., Jang Y., Tufa L.T., Gicha B.B., Lee J., Lee D., Cho B.-K. (2024). Rapid and ultrasensitive detection of thiram and carbaryl pesticide residues in fruit juices using SERS coupled with the chemometrics technique. Food Chem..

[2] Zhang Z., Zhang Q., Li L., Lin D., Jiang C. (2023). Ultrasensitive and on-site detection of carbaryl pesticides via dual-mode nanosensors utilizing portable devices. ACS Sustain. Chem. Eng..

[3] Yan H., Chen Y., Wang H., Jiao L., Chen H., Zhu C. (2023). Bismuth atom-doped gold aerogels for the detection of acetylcholinesterase activity and organophosphorus inhibitor. Chem. Eng. J..

[4] Wu J., Yang Q., Li Q., Li H., Li F. (2021). Two-dimensional MnO_2_ nanozyme-mediated homogeneous electrochemical detection of organophosphate pesticides without the interference of H_2_O_2_ and color. Anal. Chem..

[5] Fan S., Zhang G., Dennison G.H., FitzGerald N., Burn P.L., Gentle I.R., Shaw P.E. (2020). Challenges in fluorescence detection of chemical warfare agent vapors using solid-state films. Adv. Mater..

[6] Cao J., Wang M., She Y., Zheng L., Jin F., Shao Y., Wang J., El-Aty A.M.A. (2024). Highly sensitive and rapid screening technique for the detection of organophosphate pesticides and copper compounds using bifunctional recombinant TrxA-PvCarE1. J. Agric. Food Chem..

[7] Wang X., Feng T., Wang J., Hao L., Wang C., Wu Q., Wang Z. (2019). Preparation of magnetic porous covalent triazine-based organic polymer for the extraction of carbamates prior to high performance liquid chromatography-mass spectrometric detection. J. Chromatogr. A.

[8] Ruengprapavut S., Sophonnithiprasert T., Pongpoungphet N. (2020). The effectiveness of chemical solutions on the removal of carbaryl residues from cucumber and chili presoaked in carbaryl using the HPLC technique. Food Chem..

[9] Khachornsakkul K., Leelasattarathkul T. (2025). Distance-based paper analytical device for residual carbaryl pesticide quantification in food beverage samples. Sens. Actuators B Chem..

[10] Pang G., Chang Q., Bai R., Fan C., Zhang Z., Yan H., Wu X. (2020). Simultaneous screening of 733 pesticide residues in fruits and vegetables by a GC/LC-Q-TOFMS combination technique. Engineering.

[11] Hong J., Kawashima A., Hamada N. (2017). A simple fabrication of plasmonic surface-enhanced Raman scattering (SERS) substrate for pesticide analysis via the immobilization of gold nanoparticles on UF membrane. Appl. Surf. Sci..

[12] Song D., Li G., Liu Q., Huang X., Wang W., Gao F. (2025). Multi-effect coupling enhanced PtPdRhFeCu HEA/N-Cu-ZnSe@C biosensing device for pesticide residue detection in fruits and vegetables with ultra-low detection limit. Food Chem..

[13] Bilal S., Sami A.J., Hayat A., Rehman M.F.U. (2022). Assessment of pesticide induced inhibition of Apis mellifera (honeybee) acetylcholinesterase by means of N-doped carbon dots/BSA nanocomposite modified electrochemical biosensor. Bioelectrochemisty.

[14] Zhang X., Wang W., Guan L., Chen H., Li D., Zhang L., Huang S. (2024). Preparation of a Novel Green Fluorescent Carbon Quantum Dots and Application in Fe^3+^-Specific Detection in Biological System. J. Anal. Test..

[15] Wu G., Qiu H., Liu X., Luo P., Wu Y., Shen Y. (2023). Nanomaterials-based fluorescent assays for pathogenic bacteria in food-related matrices. Trends Food Sci. Technol..

[16] Liu D., Chen W., Wei J., Li X., Wang Z., Jiang X. (2012). A highly sensitive, dual-readout assay based on gold nanoparticles for organophosphorus and carbamate pesticides. Anal. Chem..

[17] Xie H., Bei F., Hou J., Ai S. (2018). A highly sensitive dual-signaling assay via inner filter effect between g-C3N4 and gold nanoparticles for organophosphorus pesticides. Sens. Actuators B Chem..

[18] Ye Q., Dai T., Shen J., Xu Q., Hu X., Shu Y. (2023). Incorporation of Fluorescent Carbon Quantum Dots into Metal–Organic Frameworks with Peroxidase-Mimicking Activity for High-Performance Ratiometric Fluorescent Biosensing. J. Anal. Test..

[19] Shi J., Tian F., Lyu J., Yang M. (2015). Nanoparticle based fluorescence resonance energy transfer (FRET) for biosensing applications. J. Mater. Chem. B.

[20] Kumar P., Kim K.-H., Deep A. (2015). Recent advancements in sensing techniques based on functional materials for organophosphate pesticides. Biosens. Bioelectron..

[21] Wu D., Zhao Q., Wang Y., Zhang B., Tang X., Talap J., Sun J., Yang X. (2024). Fluorescent Iron-Doped Polymer Dot Nanozyme-Based Cascade System for Dual-Mode Detection of Acetylcholinesterase Activity and Its Inhibitors. Anal. Chem..

[22] Mahmoudi N., Fatemi F., Rahmandoust M., Mirzajani F., Siadat S.O.R. (2023). Development of a carbon quantum dot-based sensor for the detection of acetylcholinesterase and the organophosphate pesticide. Heliyon.

[23] Zhao F., Wu J., Ying Y., She Y., Wang J., Ping J. (2018). Carbon nanomaterial-enabled pesticide biosensors: Design strategy, biosensing mechanism, and practical application. TrAC Trends Anal. Chem..

[24] Ren H., Tai S., Barimah A.O., Mao M., Peng C., Xu J., Wang Z. (2024). Advancement of pesticides fluorescence detection: From sensing strategies to application prospect. Trends Food Sci. Technol..

[25] Yang J., Liu H., Huang Y., Li L., Liu H., Ding Y. (2024). Carbon Dots as “On–Off–On” Fluorescence Sensors for Selective and Consecutive Detection of 4-Nitrophenol and Cerium(IV) in Water Samples. J. Anal. Test..

[26] Xu M., Li X., Liu P., Liu J., Han X., Chai G., Zhong S., Yang B., Cui L. (2025). A novel and visible ratiometric fluorescence determination of carbaryl based on red emissive carbon dots by a solvent-free method. Chin. Chem. Lett..

[27] Yue X., Wu C., Zhou Z., Fu L., Bai Y. (2022). Fluorescent Sensing of Ciprofloxacin and Chloramphenicol in Milk Samples via Inner Filter Effect and Photoinduced Electron Transfer Based on Nanosized Rod-Shaped Eu-MOF. Foods.

[28] Li B., Zhou M., Zhao C., Xiao L., Qi T., Xu H., Guo L., Ning G., Lu X., Zhu K. (2025). Dual-Mode Colorimetric and Fluorescent Detection of Tumor Cells Based on Gold Nanoparticles-Loaded Phosphine Covalent Organic Frameworks. J. Anal. Test..

[29] Zhai Y., Jin L., Wang P., Dong S. (2011). Dual-functional Au–Fe_3_O_4_ dumbbell nanoparticles for sensitive and selective turn-on fluorescent detection of cyanide based on the inner filter effect. Chem. Commun..

[30] Chen X., He Z., Jiao S., Sun Z., Zhang S., Liu X. (2025). Colorimetric-fluorescent dual-mode nanosensor-powered enzyme immunoassay for ochratoxin A via alkaline phosphatase-mediated silver nanoparticle growth and fluorescence inner filter effect. J. Hazard. Mater..

[31] Zheng M., Xie Z., Qu D., Li D., Du P., Jing X., Sun Z. (2013). On–off–on fluorescent carbon dot nanosensor for recognition of chromium(VI) and ascorbic acid based on the inner filter effect. ACS Appl. Mater. Interfaces.

[32] George J.K., Ramu S., Halali V.V., Balakrishna R.G. (2021). Inner filter effect as a boon in perovskite sensing systems to achieve higher sensitivity levels. ACS Appl. Mater. Interfaces.

[33] Sajwan R.K., Lakshmi G., Solanki P.R. (2021). Fluorescence tuning behavior of carbon quantum dots with gold nanoparticles via novel intercalation effect of aldicarb. Food. Chem..

[34] Khodadoust S., Nasab R.B., Zeraatpisheh F. (2024). Ultrasound-assisted dispersive nano sponge-activated carbon for extraction of propoxur and carbaryl: Response surface methodology. J. Water Process Eng..

[35] Liu Q., Fei A., Huan J., Mao H., Wang K. (2015). Effective amperometric biosensor for carbaryl detection based on covalent immobilization acetylcholinesterase on multiwall carbon nanotubes/graphene oxide nanoribbons nanostructure. J. Electroanal. Chem..

[36] Li Y., Shi L., Han G., Xiao Y., Zhou W. (2017). Electrochemical biosensing of carbaryl based on acetylcholinesterase immobilized onto electrochemically inducing porous graphene oxide network. Sens. Actuators B Chem..

[37] Shahdost-fard F., Fahimi-Kashani N., Hormozi-nezhad M. (2021). A ratiometric fluorescence nanoprobe using CdTe QDs for fast detection of carbaryl insecticide in apple. Talanta.

[38] Tian F., Jiang L., Wang Z., Peng L., Zhang Z., Huang Y. (2024). Mn^2+^-activated CRISPR-Cas12a strategy for fluorescence detection of the insecticide carbaryl. Sens. Actuators B Chem..

[39] Liu P., Li X., Xu X., Ye K., Wang L., Zhu H., Wang M., Niu X. (2021). Integrating peroxidase-mimicking activity with photoluminescence into one framework structure for high-performance ratiometric fluorescent pesticide sensing. Sens. Actuators B Chem..

[40] Wang J., Sun X., Pan W., Wang J. (2022). A fluorescence and phosphorescence dual-signal readout platform based on carbon dots/SiO_2_ for multi-channel detections of carbaryl, thiram and chlorpyrifos. Microchem. J..

